# The role of VAP-1 in cardiovascular disease: a review

**DOI:** 10.3389/fcvm.2025.1549157

**Published:** 2025-05-19

**Authors:** Chengqian Chen, Wentao Zhong, Hao Zheng, Wei Zhao, Yushi Wang, Qi Dong, Botao Shen

**Affiliations:** ^1^ Department of Cardiology Center, The First Hospital of Jilin University, Changchun, China; ^2^ Department of Endocrinology and Metabolism, The First Hospital of Jilin University, Changchun, China

**Keywords:** VAP-1, SSAO, cardiovascular diseases, biomarker, atherosclerotic

## Abstract

Cardiovascular diseases (CVD) remain the primary cause of morbidity and mortality in developed countries, highlighting the urgent need to identify biomarkers associated with CVD and its risk factors. Vascular adhesion protein-1 (VAP-1), a 170 kDa surface molecule expressed predominantly by endothelial cells, smooth muscle cells, and adipocytes, has garnered significant attention in this field. Beyond its role in inducing inflammatory mediators, VAP-1 is closely linked to coronary artery disease, heart failure, diabetes, obesity, and other CVDs, along with their associated risk factors. Notably, elevated plasma VAP-1 activity has been observed in patients with CVD and diabetes. The toxic metabolites produced by its enzymatic activity contribute to vascular endothelial injury and oxidative stress, thereby accelerating atherosclerosis and diabetes-related cardiovascular complications. Consequently, understanding the pathophysiological roles of VAP-1 in CVD has become a major research focus. This review examines the effects of VAP-1 on CVD pathogenesis and explores the therapeutic potential of VAP-1 inhibitors in managing these conditions.

## 1 Introduction

Vascular adhesion protein-1 (VAP-1), also known as semicarbazide-sensitive amine oxidase (SSAO) or primary amine oxidase, belongs to the family of copper-containing amine oxidases. This protein was first identified in 1992 as a novel adhesion molecule capable of mediating endothelial cell-lymphocyte interactions, encoded by the amine oxidase copper-containing 3 (*AOC3* gene, official gene nomenclature) (1). Notably, the SSAO family also encompasses AOC1 (encoding diamine oxidase) and AOC2 (encoding retina-specific amine oxidase), whereas VAP-1/SSAO specifically refers to the AOC3 gene product (2). SSAO is ubiquitously expressed in mammalian tissues and plasma, with interspecies variations in isoform composition, structure, and ligand specificity (3–6). Ruminants show the highest plasma SSAO activity, while human tissues display greater activity than rodent and porcine systems (7). The mature, functional form of VAP-1 is a heavily sialylated 170-kDa dimer that adopts a heart-shaped configuration, with each monomer containing three key domains (D2, D3, and D4) characteristic of dimeric proteins (8). Immunoblotting under non-reducing conditions primarily detects the 170-kDa dimer, which likely consists of two 90-kDa glycoprotein subunits, as reduction or boiling disrupts its integrity. In contrast, metabolic labeling studies also reveal a 90-kDa species, which may represent a monomeric or proteolytic fragment observed primarily in organ cultures (9, 10). Each subunit comprises an extracellular region containing the catalytic site, a transmembrane domain, and a short intracellular N-terminal tail that lacks known binding motifs, suggesting it may not play a role in signal transduction (8, 11). VAP-1 at the plasma membrane typically forms homodimers consisting of two ∼90 kD glycoproteins, with the extracellular portion of each monomer consisting of three structural domains (D2- D4) (9, 12). The D2 and D3 structural domains adopt the same *α*-*β* fold, characterized by alternating *α*-helices and *β*-strands. The large D4 domains in each subunit are catalytic domains containing residues involved in the modification of topaquinone and its localization, catalytic bases, and copper-coordinated histidine, which form the surface of the dimer and each of which also contains a catalytic site buried on the surface of the deep cleavage site (12). The catalytic activity of VAP-1/SSAO depends on key structural components, including a quinone cofactor, copper ions, and six glycosylation sites. Glycosylation, particularly near the catalytic entrance, influences substrate specificity (13). The molecule's sensitivity to oxidative agents is attributed to its incorporation of 2 mol copper ions, 1 mol carbonyl cofactors, and a 6-hydroxydopamine quinone residue per mol of protein (14).

Functionally, VAP-1 exhibits dual biological roles as both an amine oxidase and an adhesion molecule (15–17). Its oxidase activity catalyzes the oxidative deamination of short-chain primary amines, generating aldehydes, hydrogen peroxide (H2O2), and ammonia (16). In contrast, its adhesion molecule activity facilitates the selective binding of lymphocytes to vascular endothelium in a sialic acid-dependent manner (17). These dual roles are intricately linked to cardiovascular disease (CVD) pathogenesis.VAP-1 exists in one main form, a membrane-bound form, mainly on the surface of vascular endothelium, smooth muscle cells, and adipocytes (18–20). Additionally, studies have reported the presence of VAP-1 in myofibroblasts, identifying it as a novel biomarker (21). From a functional point of view, VAP-1 expressed in smooth muscle cells has SSAO activity and catalyzes exogenous and endogenous deamination of primary amines but does not have the ability to bind to lymphocytes (19). In contrast, VAP-1 localized on vascular endothelium possesses both adhesive functions and enzymatic activity (22, 23). In addition to the membrane-bound form of VAP-1, another form is soluble vascular adhesion protein-1 (sVAP-1), which is present in the blood circulation (24, 25). Membrane-bound VAP-1 is cleaved by matrix metalloproteinases (MMPs) to generate sVAP-1 (25–27). Both membrane-bound and soluble forms of VAP-1 exhibit similar enzymatic properties, sharing at least 80% sequence homology (24). However, it is believed that the membrane-bound form of VAP-1 exhibits higher activity than its soluble counterpart. The membrane-bound VAP-1 can rapidly relocate to the cell surface under acute inflammatory conditions to exert its functions, whereas sVAP-1 primarily accumulates at elevated concentrations in various chronic diseases with a relatively slower response (28, 29). Moreover, the deamination of endogenous substrates by membrane-bound VAP-1 can directly damage endothelial cells, enhance oxidative glycation, and increase oxidative stress mediators (16). In contrast, the effects of sVAP-1 are further constrained by its concentration in the bloodstream.

Elevated sVAP-1 levels have been documented in various CVDs and diabetes, including coronary artery disease, aortic stenosis, hypertension, heart failure, and stroke (30, 31). Furthermore, sVAP-1 is significantly elevated in patients with nonalcoholic fatty liver disease (NAFLD), suggesting its potential role as a pathogenic link between NAFLD and CVD (32). The widespread expression of VAP-1 within the vascular system underscores its pivotal role in inflammatory processes and its contribution to CVD pathophysiology. In the past decade, clinical studies have established VAP-1 as a novel biomarker for CVD and a promising therapeutic target. Understanding its specific mechanisms in disease progression may enable the development of innovative strategies for CVD management. This review focuses on the intricate relationship between VAP-1 and CVD, as well as the therapeutic potential of targeting VAP-1.

## 2 Pathophysiological mechanisms of VAP-1 in cardiovascular diseases

CVD represent a significant global health challenge, with atherosclerotic cardiovascular disease (ASCVD) being the primary contributor to mortality worldwide. The incidence and fatality rates of ASCVD, which encompasses coronary heart disease (CHD), ischemic stroke (IS), and peripheral vascular disease, continue to rise (33–35). Atherosclerosis, the pathological basis of ASCVD, leads to acute and chronic ischemic events due to progressive arterial lumen narrowing, plaque instability, intraplaque hemorrhage, rupture, and thrombus formation (36, 37). The growing burden of CVD, exacerbated by socioeconomic development and an aging population, has emerged as a major public health issue, with younger individuals increasingly affected (33–35). T cells, B cells, neutrophils, macrophages, and NK cells all play respective roles in CVD (particularly T cells and macrophages), and VAP-1 exerts a recruitment effect on all of them (22, 38–44). VAP-1, a multifunctional adhesion molecule, plays a crucial role in the pathogenesis of atherosclerosis. It exerts its effects through inflammation regulation, vascular endothelial damage induction, glucose and lipid metabolism modulation, and plaque stability alteration. Strong associations between VAP-1 and both the development and prognosis of ASCVD have been established in numerous studies.

**Figure 1:**
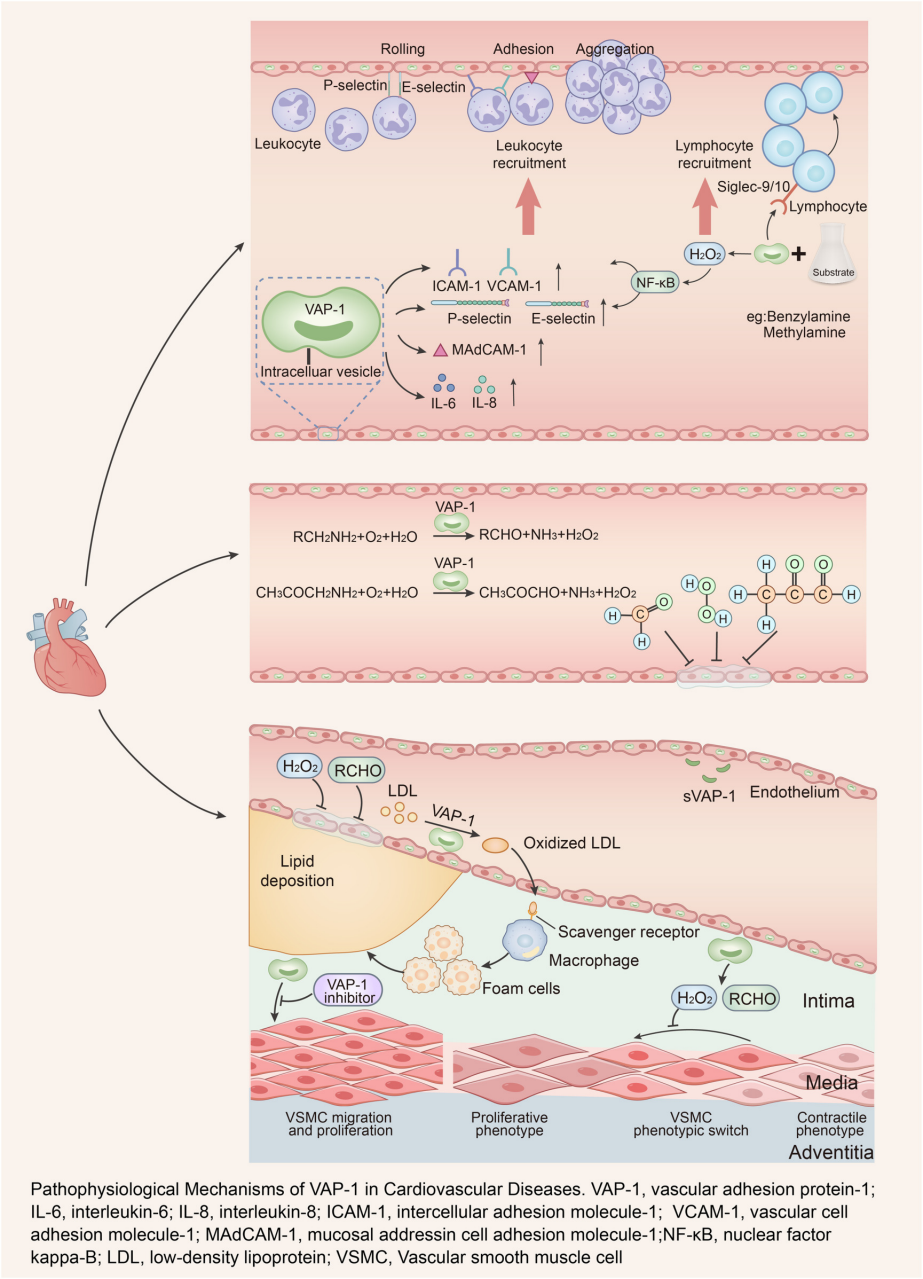
Pathophysiological mechanisms of VAP-1 in cardiovascular diseases. VAP-1, vascular adhesion protein-1; IL-6, interleukin-6; IL-8, interleukin-8; ICAM-1, intercellular adhesion molecule-1; VCAM-1, vascular cell adhesion molecule-1; MAdCAM-1, mucosal addressin cell adhesion molecule-1; NF-κB, nuclear factor kappa-B; LDL, low-density lipoprotein; VSMC, vascular smooth muscle cell.

The pathophysiological mechanisms of VAP-1 involvement in cardiovascular disease are depicted in Figure 1.

### 2.1 VAP-1 in inflammatory responses

Atherosclerosis is fundamentally a chronic inflammatory disease, characterized by highly specific cellular and molecular reactions throughout its progression—from plaque initiation to instability (45, 46). VAP-1 significantly contributes to this process by mediating leukocyte adhesion, migration, and chemotaxis, thereby amplifying vascular inflammation. As an adhesion molecule, VAP-1 interacts with Siglec-9 and Siglec-10 receptors on lymphocytes to facilitate their binding to endothelial cells (2, 42, 47). Its SSAO activity further enhances the expression of adhesion molecules, including E-selectin, P-selectin, interleukin-6 (IL-6), interleukin-8 (IL-8), intercellular adhesion molecule-1 (ICAM-1), and vascular cell adhesion molecule-1 (VCAM-1), which collectively strengthen leukocyte-endothelial interactions (48–52). Moreover, VAP-1 promotes leukocyte migration to inflammatory sites by inducing the secretion of mucosal addressin cell adhesion molecule-1 (MAdCAM-1) and establishing hydrogen peroxide gradients in the extracellular matrix (53, 54). Leukocyte surface glycoproteins, belonging to the sialic acid adhesion molecule family, serve as ligands for VAP-1, forming transient covalent bonds with its amine groups to mediate leukocyte extravasation (42, 55). Inhibition of VAP-1 has been shown to reduce granulocyte adhesion, increase rolling velocity and jump frequency, and decrease CXCL1 and MAdCAM-1 levels, thereby mitigating leukocyte recruitment and migration (22, 51, 56, 57). The above mechanisms provide a theoretical basis for VAP-1-mediated leukocyte infiltration into plaques. Jaakkola et al. reported that VAP-1 expression was significantly elevated in coronary vessels surrounding myocardial infarction regions, with increased leukocyte extravasation observed (58). Additionally, blocking endothelial VAP-1 reduced granulocyte adhesion by 60% in ischemia-reperfusion models. These findings underscore the pivotal role of VAP-1 in leukocyte trafficking during atherosclerosis.

### 2.2 VAP-1-induced vascular endothelial injury

Endothelial dysfunction, a critical initiating factor in atherosclerosis, is driven by inflammation, immune responses, and oxidative stress (59, 60). VAP-1 contributes to endothelial injury through its enzymatic activity, which catalyzes the oxidative deamination of physiological substrates such as methylamine and aminoacetone. This process generates reactive byproducts, including formaldehyde, methylglyoxal (MGO), H2O2, and ammonia (61). Tobacco smoking and adrenal hormone hypersecretion constitute established cardiovascular risk factors, both of which promote increased systemic methylamine concentrations (62, 63). Elevated concentrations or enzymatic activity of VAP-1/SSAO have been observed in patients with tobacco use, atherosclerosis, hypertension, heart failure, aortic stenosis, coronary artery disease, and diabetes mellitus, potentially leading to enhanced conversion of methylamine and aminoacetone (64–71). Formaldehyde, a highly reactive and toxic byproduct, disrupts endothelial integrity and is implicated in atherosclerosis pathogenesis (61, 72). It reacts with amines or amides to form irreversible protein-DNA crosslinks (DPCs), impairing DNA structure and function (72). Inadequate repair of these crosslinks during DNA replication can result in aberrant gene expression, contributing to endothelial dysfunction and vascular stiffening (73, 74). In mice susceptible to AS, increased methylamine deamination was found to result in a significant increase in formaldehyde production, which further supports that VAP-1-mediated production of this toxin may contribute to the development of AS (75). Similarly, VAP-1-mediated MGO production is highly cytotoxic (76). Hyperglycemia, oxidative stress, inflammation, and exogenous sources of MGO all lead to elevated MGO levels and reduced glyoxalase 1 (Glo1) activity, and an MGO-Glo1 imbalance will lead to vascular dysfunction (77). Downregulation of Glo1 allows for the perpetuation of MGO accumulation, which has been linked to age-related diseases such as diabetes mellitus, obesity, central nervous system disorders, and cardiovascular diseases that are closely associated with endothelial dysfunction (77–79). MGO reacts mainly with arginine residues on proteins, of which methylglyoxal hydroimidazolone (MG-H1) is the most common MGO-derived AGE (advanced glycosylation end product) modification found *in vivo*, which leads to structural alterations, inactivation, and degradation of the target protein (80). Thus, elevated MGO levels impair endothelial function in various ways and in different regions of the organism. Another product generated by the catalytic reaction of VAP-1, H2O2, can not only be converted to oxygen free radicals, resulting in elevated levels of oxidative stress, which in turn damages endothelial cells and contributes to the pathogenesis of a variety of cardiovascular diseases (81). In addition, H2O2 can co-modify various proteins with aldehydes and glucose to produce advanced glycated end products (AGEs) (82, 83), which are important factors leading to atherosclerosis (84). In transgenic mice with endothelial-specific overexpression of human VAP-1/SSAO, increased formation of AGEs in glomeruli and upregulation of AGE receptor expression were observed (26). Clinical studies have further confirmed that serum VAP-1 levels show significant positive correlations with systemic oxidative stress (ROS) and AGE levels in humans (85). Collectively, these mechanisms highlight the detrimental impact of VAP-1's enzymatic byproducts on vascular endothelial health, reinforcing its critical role in atherosclerosis development.

### 2.3 VAP-1 in the regulation of glucose and lipid metabolism

Disturbances in glucose and lipid metabolism are well-established major risk factors for atherosclerosis (86). The role of VAP-1 in regulating glucose uptake has been a major focus of translational medical research. Research has shown that VAP-1 substrates, such as benzylamine, combined with low-dose vanadate, improve glucose tolerance by enhancing glucose transporter-4 (GLUT-4) expression in adipocytes and reducing hyperglycemia (87). In type 2 diabetes models like Goto-Kakizaki rats, both acute and chronic administration of benzylamine (with vanadate) stimulated glucose utilization in adipocytes and muscle cells, upregulated GLUT-4 expression, and alleviated insulin resistance in muscle tissue (88). *In vitro* experiments using liver slice models developed by Karim et al. (89) demonstrated an oxidase activity-dependent increase in both glucose uptake and GLUT-4 expression. However, transgenic mice overexpressing VAP-1 initially showed improved glucose tolerance, which was later offset by vascular complications typical of diabetes, such as glomerulosclerosis, atherosclerosis, and hypertension (26). Clinical studies have corroborated a positive correlation between fasting sVAP-1 levels and fasting blood glucose. Elevated sVAP-1 levels have also been observed during oral glucose tolerance tests, with levels peaking two hours after glucose loading, further correlating with fasting sVAP-1 levels (90). In cholesterol-rich diet-fed KKAy diabetic mice, SSAO inhibitors reduced body weight and atherosclerotic lesions (91, 92). Interestingly, VAP-1 knockout mice exhibited mild obesity and reduced leukocyte infiltration in adipose tissue but maintained normal blood glucose and glucose tolerance (93). These findings imply a role for VAP-1 in glucose regulation, though its precise mechanisms remain uncertain. One study proposed that VAP-1 exerts insulin-like effects by inducing Caveolin-1 mRNA expression and catalyzing endogenous amines to produce hydrogen peroxide. This activity was absent in adipocytes from AOC3 knockout mice (94–96). While elevated sVAP-1 levels under hyperglycemic conditions might confer hypoglycemic effects, conflicting data exist. For instance, studies using the VAP-1 inhibitor PXS-4728A reported reduced blood glucose levels in apolipoprotein E-deficient mice after 7 and 15 weeks of treatment (70). Therefore, the precise mechanisms underlying VAP-1-mediated regulation of blood glucose levels remain to be fully elucidated.

VAP-1 also plays a role in lipid metabolism. Hydrogen peroxide, a key product of its catalytic reactions, generates ROS (97, 98). These ROS oxidatively modify low-density lipoprotein (LDL), resulting in dysfunctional LDL that bypasses normal metabolic pathways and is absorbed by scavenger receptors, leading to foam cell formation and lipid accumulation within vascular walls (99–103). Metabolites of VAP-1 lead to endothelial cell damage, lipid deposition in the vessel wall, and ultimately, the formation of atherosclerotic lesions. A large clinical study found that MACROD2 gene expression could promote VAP-1 production in adipose tissue, and its level was positively correlated with body mass index (104), and higher levels of VAP-1 were also observed in obese mice, suggesting that obesity and related genetic factors may stimulate the production of VAP-1 in adipose tissue (94). VAP-1 inhibitors have demonstrated effects such as suppressed adipogenesis, reduced body weight, and lower cholesterol levels in mice. Conversely, AOC3 knockout mice showed increased weight, fat mass, and total cholesterol levels (96, 105–108). In addition, VAP-1 has been found to be negatively correlated with HDL cholesterol levels (104, 109). Notably, Weston et al. (32) demonstrated that genetic ablation of AOC3 attenuated the severity of hepatic steatosis in mice, whereas expression of enzymatically inactive VAP-1 failed to produce this protective effect. These results suggest that VAP-1 plays a multifaceted role in lipid metabolism, though further exploration is necessary. In summary, VAP-1 significantly influences glucose and lipid metabolism, both of which are integral to atherosclerosis development. However, its precise regulatory mechanisms remain to be fully elucidated.

### 2.4 VAP-1 in atherosclerosis

Vascular smooth muscle cells (VSMCs) play a critical role in maintaining atherosclerotic plaque stability. Reductions in VSMC numbers or phenotypic changes directly contribute to atherosclerosis progression (110–112). Current evidence suggests that inhibition of VAP-1 reduces monocyte adhesion and transendothelial migration, suppresses macrophage recruitment and activation, and decreases smooth muscle cell (SMC) migration and proliferation, thereby significantly attenuating the formation or progression of atherosclerotic lesions (70, 108). Studies in VAP-1 knockout mice have demonstrated increased VSMC content within plaques and a phenotypic shift from contractile to synthetic states, resulting in higher collagen deposition. This structural remodeling produces thicker fibrous caps, improving plaque stability (31, 113–115). Similarly, semicarbazide treatment in LDL receptor knockout (LDLr^−/−^) mice for 6–9 weeks yielded comparable improvements in plaque composition (113). Further research using PXS-4728A in cholesterol-fed rabbit models of atherosclerosis demonstrated that 12-week treatment reduced VSMC migration and proliferation (108). Discrepancies in study outcomes may stem from differences in animal models, inhibitor selection, or treatment durations. Overall, these findings underscore the therapeutic potential of targeting VAP-1 to improve atherosclerotic plaque stability and mitigate associated cardiovascular risks.

## 3 VAP-1 and coronary heart disease (CHD)

A large prospective cohort study demonstrated that elevated sVAP-1 levels are strongly associated with an increased risk of major adverse cardiovascular events (MACE), including CHD, unstable angina, acute myocardial infarction, coronary revascularization, and stroke (109). Serum VAP-1 independently predicts 10-year all-cause mortality, cardiovascular mortality, and cancer mortality in subjects with type 2 diabetes (116). Additionally, elevated sVAP-1 levels were linked to increased mortality risk. These findings suggest that plasma VAP-1 serves as a reliable biomarker for CHD presence and severity. Furthermore, inhibition of VAP-1 activity, such as through PXS-4728A, has been proposed as a potential therapeutic strategy for atherosclerosis management (31, 70). Clinical studies have consistently shown higher plasma VAP-1 levels in CHD patients compared to healthy controls, with a positive correlation between sVAP-1 concentrations and CHD severity (70). Elevated sVAP-1 levels were also associated with both the number and extent of coronary artery stenoses. Even after adjusting for confounding factors, sVAP-1 levels remained an independent predictor of CHD severity (OR = 2.09, 95% CI: 1.29–3.38; *P* = 0.003) (70). This highlights the potential of VAP-1 as a diagnostic and prognostic tool for CHD. Preclinical studies further validate the therapeutic potential of VAP-1 inhibition. In apolipoprotein E-deficient mice, VAP-1/SSAO inhibitors significantly reduced atherosclerotic plaque area in both preventive and therapeutic regimens. For instance, 15 weeks of PXS-4728A treatment alongside a high-fat diet reduced plaque formation to a degree comparable to atorvastatin (2.5 mg·kg^−1^·d^−1^ for 15 weeks) (70). These findings suggest that VAP-1 inhibitors may represent a novel class of therapeutics for CHD. The association between VAP-1 and calcific aortic stenosis (CAS) also underscores its clinical relevance. In patients undergoing aortic valve replacement, elevated VAP-1 mRNA expression was observed in calcified regions of the aortic valve (117, 118). Plasma VAP-1 levels were found to increase proportionally with CAS severity (69, 118), indicating its potential role in the progression of valvular diseases. Beyond CHD, VAP-1 has been implicated in hyperglycemia-induced atherosclerosis. Studies in low-risk populations have identified plasma VAP-1 concentrations as predictors of carotid intima-media thickness (IMT) and plaque formation (109, 119). Karadi et al. demonstrated that serum SSAO activity correlates with carotid artery stenosis severity and plaque scores in diabetic patients (120). In non-diabetic subjects, glucose loading during oral glucose tolerance tests increased sVAP-1 levels, which were independently associated with serum AGEs and carotid IMT (85). Aalto et al. conducted a longitudinal study involving 2,138 healthy individuals and reported a positive correlation between sVAP-1 activity, IMT, and carotid plaque formation. This suggests a potential role for sVAP-1 in subclinical atherosclerosis development (121). The therapeutic implications of VAP-1 inhibition extend beyond reducing atherosclerotic lesion size and inflammation to stabilizing plaques and preventing adverse clinical events. For example, in a rat model of myocardial ischemia-reperfusion injury, elevated myocardial SSAO activity was associated with increased leukocyte infiltration, endothelial P-selectin expression, and oxidative stress markers. Treatment with SSAO inhibitors—semicarbazide (a non-specific agent concurrently inhibiting both SSAO and Lysyl Oxidase), hydralazine (an irreversible SSAO inhibitor with additional inhibitory effects on aldehyde oxidase and NADPH oxidase), and LJP 1207 (a selective SSAO inhibitor)—significantly ameliorated the aforementioned pathological alterations and reduced infarct size (122). These findings emphasize the critical role of VAP-1/SSAO in CHD development and post-myocardial infarction tissue damage. While immune and inflammatory mechanisms are well-established contributors to CHD progression, most anti-inflammatory therapies have failed to demonstrate significant benefits in clinical trials. This highlights the need for precise inflammatory targets. Current evidence positions VAP-1 as a promising therapeutic target for CHD, offering new avenues for disease management and prevention.

## 4 VAP-1 and essential hypertension

Essential hypertension, a common cardiovascular disease among middle-aged and elderly individuals, is influenced by both environmental and genetic factors. It is a significant risk factor for severe clinical events such as coronary heart disease, heart failure, stroke, and end-stage renal disease. The disease is characterized by a high prevalence, insidious onset, low awareness rate, high disability rate, and the need for lifelong medication (123). Although the exact pathogenesis of essential hypertension remains unclear, most studies suggest it involves a chronic low-grade inflammatory process (124, 125). Inflammatory responses disrupt vascular microenvironment homeostasis by driving monocyte and macrophage infiltration during vascular injury. This triggers the secretion of adhesion molecules and other vascular active substances, promoting endothelial cell proliferation, migration, and differentiation. Such pathological vascular remodeling processes are integral to the development, progression, and complications of hypertension (126–128). Previous studies have suggested that inflammatory response increases the prevalence of hypertension and is closely related to the diagnosis, treatment, and prognosis of patients with hypertension, making it an important area of research in the pathogenesis of hypertension (129). Meanwhile, recent studies have shown that the increase in the levels of inflammatory factors precedes the increase in clinical blood pressure levels, which may have potentially important clinical applications in the prediction and diagnosis of hypertension (130). Maciorkowska et al. reported elevated circulating renin and VAP-1 levels in hypertensive patients with poorly controlled blood pressure (67). As a marker of systemic inflammation, VAP-1 is implicated in various inflammatory pathways (119, 131). Inhibition of VAP-1 has been shown to reduce levels of inflammatory mediators such as ICAM-1, MCP-1, and TNF-α, thereby alleviating inflammation (132). These findings suggest that VAP-1 may contribute to the development of hypertension via inflammatory mechanisms, potentially influencing its prevalence, diagnosis, and treatment. Additionally, excessive SSAO activity associated with VAP-1 may affect vascular elasticity by increasing elastin cross-linking and altering the structure of newly synthesized elastin. However, studies involving SSAO-deficient arteries have shown conflicting results. For instance, SSAO^−/−^mice exhibited increased arterial diameter with mechanical properties comparable to those of normal arteries, challenging the hypothesis that SSAO significantly alters elastic fiber organization and vascular responsiveness (27, 133). Overall, there is a strong correlation between VAP-1 and essential hypertension, likely mediated by inflammatory processes. Pharmacological targeting of VAP-1 may offer a novel approach to reducing inflammation and slowing hypertension progression. However, further research is needed to fully elucidate the role of VAP-1 in hypertension pathogenesis and to develop targeted therapeutic strategies.

## 5 VAP-1 and obesity

Obesity, a major risk factor for cardiovascular diseases, is closely linked to metabolic disorders such as diabetes and dyslipidemia (134–136). VAP-1 has garnered significant attention in obesity research due to its insulin-like effects and high expression on adipocyte membranes. In adipocytes, VAP-1 colocalizes with GLUT-4 within endosomal compartments, facilitating glucose uptake by stimulating GLUT-4 translocation to the plasma membrane (88, 137). VAP-1 enzymatic byproducts, including hydrogen peroxide, enhance glucose uptake and inhibit adipogenesis when combined with SSAO substrates and vanadate (138). These effects mimic insulin action. Jargaud et al. demonstrated that AOC3 knockout (AOC3KO) and VAP-1 mutant (AOC3KI) mice, which lack functional SSAO activity, exhibited greater fat deposition than controls, despite normal food intake (96). Thus, adipocyte-expressed VAP-1 deserves more attention in improving blood glucose and controlling obesity. While AOC3KO mice showed mild obesity (96), other studies reported a negative correlation between circulating sVAP-1 levels and obesity (90). Conversely, serum VAP-1 activity has been positively associated with body mass index (BMI) (139, 140). Increased SSAO activity in adipose tissue has been linked to low-grade inflammation observed in obesity and diabetic obesity (137). However, to date, there is no conclusive evidence of a link between VAP-1 and obesity. Current evidence indicates that VAP-1 expression is significantly upregulated in differentiated adipocytes compared to preadipocytes, paralleling an increase in enzymatic activity (20). In certain obese individuals, membrane-bound VAP-1 may remain localized within adipose tissue rather than being cleaved into circulation, potentially contributing to adipocyte proliferation and glucose transport while reducing circulating sVAP-1 levels (93, 121). However, the mechanisms governing sVAP-1 shedding and its regulation remain unclear and may depend on the severity of obesity and its associated comorbidities. Further research is required to elucidate the complex role of VAP-1 in obesity pathophysiology, particularly its contributions to adipose tissue inflammation and metabolic dysfunction.

## 6 VAP-1 and diabetes

Elevated plasma SSAO levels and activity are strongly associated with the onset and progression of diabetes. SSAO catalyzes the oxidative deamination of endogenous substrates such as methylamine and aminoacetone, producing toxic metabolites that directly damage vascular endothelial cells, promote glycation, and enhance oxidative stress. These processes exacerbate diabetes and contribute to its vascular complications. The primary mechanisms by which SSAO/VAP-1 influences diabetes pathogenesis involve oxidative stress and the formation of AGEs. Increased SSAO activity is closely linked to late-stage complications of diabetes, including atherosclerosis, retinopathy, and nephropathy. The link between sVAP-1 and diabetes was initially identified in individuals with type 1 diabetes (141, 142). sVAP-1 levels and SSAO activity were higher in individuals with type 1 diabetes, and plasma sVAP-1 was positively correlated with blood glucose (141, 142). Subsequently, plasma sVAP-1 was also found to be elevated in studies of type 2 diabetic patients compared to normal subjects (143). In addition, it has been shown that in patients with gestational diabetes, sVAP-1 levels are higher than in normoglycemic pregnant women (144). In the Taiwan Lifestyle Cohort Study, prediabetic individuals were found to have higher serum sVAP-1 levels compared to normoglycemic controls, further implicating sVAP-1 as a potential biomarker for early metabolic dysregulation (90). VAP-1 is also linked to diabetes complications such as retinopathy and nephropathy. Plasma VAP-1 levels correlate positively with vascular endothelial growth factor (VEGF) levels, which are elevated in diabetic retinopathy (132). Several studies suggest that VAP-1 is associated with the pathogenesis of diabetic retinopathy (30, 145, 146). Animal studies testing VAP-1 inhibitors have demonstrated therapeutic potential. For instance, RTU-1096 prevented retinal thickening in mice following laser photocoagulation, while the oral VAP-1 inhibitor 1H-imidazol-2-amine significantly reduced ocular permeability in diabetic rats (51, 147). In addition, several other studies have shown that VAP-1 is associated with the development of diabetic nephropathy (148–150). The VAP-1 inhibitor ASP8232 significantly reduced albuminuria in a phase 2 trial involving diabetic nephropathy patients (151). In patients with type 2 diabetes, we found that serum VAP-1 levels predicted the incidence of end-stage renal disease (ESRD). After adjusting for other risk factors, each standard deviation increase in serum VAP-1 was associated with a hazard ratio (HR) of 1.55 for ESRD risk (148). Beyond vascular complications, Valente et al. reported that VAP-1 levels were significantly elevated in hippocampal vessels of diabetic patients with Alzheimer's disease compared to those with Alzheimer's alone. This was accompanied by increased markers of oxidative stress, AGEs, and inflammation (152). Additionally, acute fluctuations in plasma VAP-1 levels have been observed in non-diabetic individuals during oral glucose tolerance tests, where SSAO/VAP-1 levels rose significantly 30 min post-glucose loading and remained elevated for 2 h. These changes correlated with systemic oxidative stress, AGEs, and carotid IMT, suggesting that VAP-1 could serve as a biomarker for hyperglycemia-induced atherosclerosis (85). These findings highlight the critical role of VAP-1 in diabetes onset and progression through its contributions to inflammation, oxidative stress, and AGE production. Targeting VAP-1 with inhibitors represents a promising therapeutic approach for diabetes and its complications.

## 7 VAP-1 and heart failure (HF)

HF, the terminal stage of many cardiovascular diseases, remains a leading cause of mortality worldwide. Epidemiological studies report high global prevalence and fatality rates for HF (153). In patients with chronic HF, Boomsma et al. found elevated plasma SSAO levels, with further increases observed in those with diabetes or more severe disease. These findings suggest that plasma SSAO may serve as a useful biomarker for assessing HF severity and prognosis (64). In a subsequent 3.4-year follow-up study of 372 patients, baseline plasma SSAO levels were significantly higher in those who died during the study period compared to survivors. This supports the role of SSAO as a prognostic marker for mortality in chronic HF (68). Similarly, Marinho et al. evaluated SSAO and monoamine oxidase (MAO) activity in patients with hypertensive heart disease and left ventricular systolic dysfunction (across NYHA HF classes II–IV). Both SSAO and MAO activity were significantly higher in patients than in controls, with the highest SSAO levels observed in NYHA class IV patients. These findings suggest that amine oxidases contribute to endothelial damage in HF pathogenesis (154). In addition, results from a prospective multicenter cohort study of patients undergoing hemodialysis showed that higher VAP-1 plasma levels were strongly associated with an increased likelihood of myocardial infarction, heart failure, cerebral infarction, cerebral hemorrhage, and other cardiovascular events (155). The role of VAP-1 in endothelial injury mechanisms during HF progression is particularly notable. Endothelial activation, especially in HF with preserved ejection fraction (HFpEF), plays a critical role in clinical HF. Therapies targeting endothelial activation may help prevent the progression of cardiovascular risk factors into overt HF (156). Given its involvement in leukocyte adhesion and inflammation, VAP-1 inhibition holds promise for improving HF prognosis. Despite strong evidence supporting its diagnostic and prognostic value, the specific mechanisms through which VAP-1 contributes to HF remain underexplored. Future research should investigate the therapeutic potential of VAP-1 inhibitors to better manage HF and enhance patient outcomes.

## 8 Potential of VAP-1 inhibitors as emerging therapeutics

Elevated VAP-1 activity is closely associated with the onset and progression of various diseases. By inhibiting VAP-1 activity, the oxidative deamination process can be suppressed, reducing the production of toxic metabolites and thereby minimizing endothelial damage, oxidative stress, and AGE formation. Research has shown that VAP-1 inhibition effectively decreases leukocyte extravasation and tissue inflammation (2, 56, 157, 158). VAP-1 inhibitors and anti-VAP-1 antibodies have demonstrated promising therapeutic potential in various animal models and inflammatory diseases. VAP-1's involvement in the pathogenesis of numerous conditions, such as rheumatoid arthritis, chronic liver inflammation and fibrosis, neuroinflammatory diseases, Parkinson's disease, Alzheimer's disease, and cancer, highlights its importance as a therapeutic target in the pharmaceutical industry (131, 159–164). Among small-molecule VAP-1 inhibitors, ASP8232 has shown favorable safety and tolerability profiles in phase II trials for diabetic nephropathy and diabetic macular edema (151, 165). However, its efficacy for macular edema was limited, leading to the trial's termination (165). Conversely, ASP8232 demonstrated greater promise in the ALBUM study by reducing albuminuria in diabetic nephropathy patients (151). Other VAP-1 inhibitors, such as MDL-72974A and aminoguanidine, have been shown to prevent obesity and atherosclerosis in KKAy mice (91, 92). PXS-4728A is a novel, orally available small-molecule inhibitor of VAP-1/SSAO that demonstrates irreversible and highly selective characteristics (5). It effectively suppresses neutrophil migration during acute pulmonary inflammation, lung infection, and airway hyperresponsiveness, thereby inhibiting airway inflammation and fibrosis while improving pulmonary function (57, 166). In addition to its potential therapeutic effects on atherosclerosis as described in Section 2.4, PXS-4728A has also been shown to significantly ameliorate renal fibrosis, with particularly prominent effects in diabetic nephropathy (70, 108, 167, 168). In myocardial ischemia-reperfusion injury models, SSAO inhibitors (e.g., SCZ, HYD, LJP 1207) administered shortly before reperfusion significantly reduced SSAO activity, disrupted leukocyte-endothelial adhesion, decreased leukocyte infiltration, and mitigated myocardial injury (122). However, certain VAP-1 inhibitors exert additional cardiovascular effects. For instance, hydralazine has been shown to reduce blood pressure at higher doses (169). Despite these advances, clinical trials evaluating VAP-1-targeted therapies for hypertension, coronary heart disease, heart failure, or other cardiovascular conditions are lacking. Further research is needed to explore these therapeutic possibilities. Ongoing efforts to develop high-efficiency, selective, and low-toxicity VAP-1 inhibitors are crucial for translating these findings into clinical practice. Future studies should focus on investigating novel VAP-1 inhibitors for the treatment and prevention of cardiovascular diseases.

## 9 Conclusion and future perspectives

Plasma VAP-1 levels and activity are markedly elevated in conditions such as inflammation, diabetes, atherosclerosis, and congestive heart failure. VAP-1 contributes to atherosclerosis progression through its enzymatic activity, facilitating leukocyte transendothelial migration and producing toxic metabolites that damage vascular endothelial cells. These functions position VAP-1 as a valuable biomarker for cardiovascular disease diagnosis and prognosis, aiding in the prediction of hypertension and coronary heart disease risk, as well as the assessment of major adverse event rates in CHD and HF patients. As a glycoprotein that mediates leukocyte migration and is readily detectable in circulation, VAP-1 holds significant potential as a future biomarker for cardiovascular diseases. Extensive research has explored its tissue distribution, substrates, and inhibitors, identifying it as a viable therapeutic target across multiple pathological contexts. However, the precise mechanisms underlying VAP-1's pathophysiological roles, along with the pharmacological effects of its inhibitors, remain insufficiently understood. Addressing these knowledge gaps will be critical for advancing VAP-1-targeted therapies. The development of novel, high-efficiency, and specific VAP-1 inhibitors will be instrumental in improving cardiovascular disease outcomes. Continued research efforts are necessary to fully elucidate the pathological significance of VAP-1 and to harness its therapeutic potential in future clinical applications.
